# Awareness, knowledge, and cervical cancer screening behaviour in an urban informal settlement: Evidence from multinomial and interaction models

**DOI:** 10.1371/journal.pgph.0007013

**Published:** 2026-08-21

**Authors:** Winfred Kananu Kubai, Alfred Owino Odongo, Gilbert Koome Rithaa

**Affiliations:** 1 Department of Epidemiology and Biostatistics, Mount Kenya University, Thika, Kenya; 2 Department of Public and Global Health, College of Health Sciences, University of Nairobi, Nairobi, Kenya; University of Cape Town, SOUTH AFRICA

## Abstract

Cervical cancer screening uptake in Kenya remains far below global targets, particularly in urban informal settlements where knowledge gaps persist despite widespread awareness campaigns. Understanding how knowledge shapes screening behavior is critical to explaining the persistent gap between willingness and action. The objective was to examine knowledge-related determinants of cervical cancer screening uptake among mothers aged 25–49 years in Kiandutu Informal Settlement, focusing on both main knowledge effects and interaction effects with awareness. A cross-sectional survey was conducted among 385 women. Screening behaviour was classified into three categories: Screened, Not Screened but Intends, and Not Screened, No Intention. Knowledge was measured using a composite index, and multinomial logistic regression, including interaction terms, was used to assess how knowledge and awareness jointly influence screening. Screening uptake was 48.8%. Only 18.2% of women with low knowledge had ever screened, and 62.1% had no intention to screen. Higher knowledge levels were strongly associated with screening (χ²(4) = 58.780, p < 0.001). Low knowledge independently predicted non-intention (AOR = 13.091, 95% CI: 5.526–31.013). The interaction model revealed a distinctive pattern: awareness without adequate knowledge sharply reduced intention to screen (Low Knowledge × Awareness AOR = 0.024, 95% CI: 0.003–0.219). Screening behaviour in informal settlements is shaped not by awareness alone but by the depth and accuracy of knowledge. Women require actionable understanding, not just exposure to health messages, to progress from awareness to screening intention and uptake. These findings highlight the importance of knowledge-dependent effects in cervical cancer screening behaviour.

## Introduction/Background

Cervical cancer remains a major public health burden worldwide. In 2022, an estimated 660,000 new cases and 350,000 deaths occurred globally, with most deaths concentrated in low- and middle-income countries, particularly in Sub-Saharan Africa, reflecting persistent inequities in vaccination, screening, and treatment access [1]. Persistent infection with high-risk human papillomavirus (HPV) causes nearly all cervical cancers, and women living with human immunodeficiency virus (HIV) face a six-fold higher risk of developing the disease owing to immunosuppression [1].

Despite preventability, screening coverage remains low across the region. A systematic analysis of population-based surveys in 28 Sub-Saharan African countries estimated that only ~14% of women aged 30–49 had ever been screened by 2020 (Yang et al., 2023). In Kenya, recent analyses using the Kenya Demographic Health Survey (KDHS) 2022 indicate a lifetime screening prevalence of about 16–17%, far below the WHO target of 70% [2,3]. Health system assessments further show Kenya’s screening offer is dominated by Visual Inspection with Acetic Acid (VIA) and that Human Papillomavirus (HPV) testing capacity remains limited in public facilities, underscoring delivery constraints in routine care [4].

In this study, screening is defined as proactive testing of asymptomatic women using validated methods (HPV DNA testing, cytology, or VIA) to identify precancerous lesions at an early, treatable stage, consistent with WHO guidance [1]. Awareness denotes basic recognition (e.g., having heard of cervical cancer/screening), typically captured by single-item prompts in population surveys [5]. Knowledge refers to accurate, actionable understanding of causation (HPV), prevention (including vaccination), eligibility/procedures, and the benefits of early detection. Systematic evidence across Africa shows that higher knowledge, perceived risk, and provider advice are consistently associated with screening uptake [6]. Yet, awareness often remains high while uptake stays low, suggesting knowledge may not operate in isolation, a pattern echoed in informal settlement studies and national datasets [2,5].

Three gaps in the literature motivate our study. First, most empirical studies treats awareness and knowledge as independent, additive predictors, rarely testing whether the effect of awareness depends on knowledge [6]. Second, the dominant binary outcome (ever screened vs never) obscures meaningful gradients—particularly distinguishing women who intend to screen from those with no intention [5]. Third, there is scant empirical testing of whether a minimum actionable knowledge level is required before awareness translates into intention, despite regional evidence that message clarity and information source are linked to screening among women [7,8].

In addition to screening, Kenya has adopted HPV vaccination as a key component of cervical cancer prevention in line with the WHO cervical cancer elimination strategy and its 90–70–90 targets [9]. The national HPV vaccination program was introduced in 2019, targeting adolescent girls through school-based and health facility delivery platforms. Recent efforts have focused on improving vaccine uptake through community mobilization, public awareness campaigns, and integration with adolescent health services [10]. Despite these advances, screening remains essential for women beyond the vaccination target age group and for reducing the burden of cervical cancer among adult women [10,11].

To address these gaps, we examine whether the relationship between awareness and screening behaviour varies according to women’s level of actionable knowledge. Specifically, we test whether awareness and knowledge interact in shaping screening intentions and uptake, rather than operating as independent predictors. We operationalized knowledge using a composite index and explicitly modelled knowledge–awareness interaction terms within a multinomial framework that captures screened, not screened but intends, and not screened with no intention.

This approach allows us to investigate whether the influence of awareness differs across levels of knowledge and provides insight into the behavioural pathway linking information exposure to screening behaviour [6]. This approach complements ongoing policy efforts that emphasize transitioning to high-performance testing and improving communication quality to meet the WHO 90-70-90 elimination targets [12]. The aim of this study was to examine how knowledge and awareness jointly influence cervical cancer screening behaviour among women in an urban informal settlement and to determine whether the effect of awareness differs across levels of knowledge.

## Methods

### Ethics Statement

Ethical approval was obtained from the Mount Kenya University Ethical Review Committee (MKU/ISERC/4881). A research permit was granted by the National Commission for Science, Technology and Innovation (NACOSTI/P/25/418078), and administrative approval was obtained from the Kiambu County Government (KIAMBU/HRDU/JWK/01).

Written informed consent was obtained from all participants before data collection. Consent information was provided in either English or Kiswahili, and participants were informed about the study objectives, study procedures, potential risks and benefits, confidentiality protections, and their right to decline participation or withdraw at any time without penalty. Only women aged 25–49 years were eligible for participation; no minors were enrolled in the study. Privacy was maintained through interviews conducted in private settings. All data were de-identified prior to analysis, and access to study data was restricted through role-based data management procedures to ensure confidentiality and participant protection.

As part of participant benefit and ethical good practice, all respondents were provided with standardized cervical cancer education and information regarding available screening services following completion of the interview.

### Study design and setting

This was an analytical, community-based cross-sectional study conducted between 10/05/2025 and 27/05/2025 in Kiandutu Informal Settlement, Thika, Kiambu County, Kenya. The site contains ten densely populated villages characterized by poverty, informal employment, limited health infrastructure, and persistent barriers to preventive screening. These conditions provided an appropriate context for examining the role of knowledge and awareness in cervical cancer screening behaviour among women of reproductive age.

### Sampling and participants

A multi-stage sampling approach was used. Five villages were randomly selected from the settlement’s ten administrative clusters. Within these villages, Community Health Promoters (CHPs) supported generation and verification of a household listing that served as the sampling frame. Households were selected using systematic random sampling with proportional allocation to village size. In households with more than one eligible woman, a simple random draw identified the respondent.

Eligibility criteria included women aged 25–49 years because this age group constitutes the primary target population for cervical cancer screening under Kenya National Cancer Screening Guidelines and aligns with WHO recommendations for cervical cancer prevention and early detection, residency in Kiandutu for ≥ six months, and capacity to provide informed consent. Pregnant women, those with serious comorbidities or hospitalization, and those unable to consent were excluded.

A total of 385 women participated, based on a 50% assumed prevalence, 95% confidence level, and 5% absolute precision estimated using Fischer’s formula (p = 0.5, 95% CI).

### Data collection

Five trained CHPs administered a structured questionnaire through Open Data Kit (ODK) digital application, which incorporated embedded prescreening, automated skip patterns, and real-time validation to minimise missing data. The tool assessed demographic characteristics, awareness, knowledge, and screening practices.

The questionnaire was developed from established cervical cancer screening literature and reviewed by two public health experts with experience in cancer prevention and screening research to assess content validity, clarity, and relevance. The tool was subsequently pretested among 25 women in Makongeni Informal Settlement, a community with characteristics comparable to the study setting. Feedback from the pretest was used to refine question wording, sequencing, and cultural appropriateness prior to data collection. Internal consistency of the knowledge scale was acceptable (Cronbach’s α = 0.81).

Daily uploads to an encrypted cloud folder prevented data loss while the analysis dataset was de-identified by removing direct identifiers and storing the linkage key separately under restricted access.

Following completion of each interview, all participants received standardized cervical cancer health education delivered by the trained Community Health Promoters. The information covered cervical cancer risk factors, prevention, recommended screening age, available screening services, and the benefits of early detection. This information was provided to all participants irrespective of their knowledge level, awareness status, or previous screening history.

### Measures and operational definitions

In line with WHO guidance and Kenya’s National Cancer Screening Guidelines, screening was defined as proactive testing of asymptomatic women using validated methods (HPV DNA testing, cytology, or VIA) to detect precancerous lesions at an early, treatable stage. Screening uptake was defined using three mutually exclusive categories derived from two items on screening history and intention: Screened, Not screened but intends, and Not screened, no intention. Awareness was defined as prior exposure to any information about cervical cancer.

Knowledge was measured using a five-item index (score range 0–5) assessing understanding of early detection, curability, vaccination, universal female risk, and correct screening age. Knowledge categories were defined a priori to reflect meaningful levels of actionable understanding relevant to screening behaviour. Scores of 0–1 were classified as low knowledge, indicating limited understanding of key cervical cancer concepts; scores of 2–3 were classified as moderate knowledge, reflecting partial understanding; and scores of 4–5 were classified as high knowledge, representing comprehensive knowledge across most domains assessed. Categorization was undertaken to facilitate interpretation of knowledge-related differences in screening behaviour while maintaining adequate sample sizes for multinomial and interaction analyses (Table 1).

**Table 1:** Operational definitions and description of key variables.

Construct	Definition & coding	Rationale/ Source
Screening status (primary outcome)	Three mutually exclusive categories constructed from two items: Screened (ever screened), Not screened but intends, Not screened, no intention.	Preserves behavioral patterns from action to readiness to resistance while avoiding information loss from dichotomisation.
Awareness	Binary: “Yes” if respondent had heard of cervical cancer; otherwise “No”.	Standard in population surveys and Demographic Health Survey (DHS) derived studies.
Knowledge index (0–5)	Sum of correct responses to five items: early detection facilitates treatment; cancer is curable if found early; vaccination prevents cervical cancer; false that screening age is 21–29 (reverse-scored to reflect Kenya’s 25–49 guidance); universal female risk.	Measures actionable knowledge and aligns to national/WHO guidance.
Knowledge levels	Low (0–1), Moderate (2–3), High (4–5) representing limited, partial, and comprehensive actionable knowledge.	Facilitates interpretation of knowledge-related differences in screening behaviour and supports interaction analyses.
Interaction terms	Knowledge level versus Awareness, modelled via product terms with High knowledge versus Aware as reference.	Examines whether the effect of awareness varies across knowledge levels.
Covariates	Age (25–29; 30–34; 35–44; 45–49), education (none/primary/ secondary/tertiary), employment (unemployed/self-employed/formal), income (collapsed to reduce sparsity).	A priori confounders retained parsimoniously; health-system factors were excluded by design to maintain the knowledge-only focus.

### Statistical analysis

All analyses were conducted using Statistical Package for the Social Sciences (SPSS) version 27. The analysis proceeded in several structured steps to ensure clarity, transparency, and robustness of the modelling strategy. First, descriptive statistics were used to summarise participants’ sociodemographic characteristics, levels of awareness, distribution of knowledge scores, and the three screening outcome categories. The results are reported as frequencies and percentages.

Second, we examined the crude association between each independent variable and cervical cancer screening behaviour using Pearson’s chi-square (χ^2^) test of independence. Cramer’s V was used to quantify the strength of these associations, allowing assessment of how strongly variables such as knowledge level, awareness, age, education, or income were related to screening status.

Because the outcome comprised three behaviourally distinct categories comprising screened, not screened but intends, and not screened with no intention, we employed multinomial logistic regression as the primary modelling approach. “Screened” served as the reference category. All multinomial odds ratios were therefore interpreted relative to the screened group.

Two models were fitted. Model 1, the main-effects model, included knowledge level, awareness, and prespecified sociodemographic covariates. This model provided baseline estimates of the independent effect of each variable on screening behaviour. Model 2, the interaction model, extended Model 1 by including knowledge × awareness interaction terms to assess whether the relationship between awareness and screening behaviour differed according to women’s level of actionable knowledge.

Model adequacy was evaluated using likelihood ratio chi-square tests comparing each full model against its intercept-only counterpart. Model 1 significantly improved model fit relative to the intercept-only model (χ^2^(18) = 81.34, p < 0.001), demonstrating that the included variables collectively explained variation in screening behaviour. Pseudo-R^2^ indices indicated satisfactory fit for a behavioural model. Model 2 showed further improvement (χ^2^(22) = 95.65, p < 0.001), with increased pseudo-R^2^ values and a reduction in −2 log likelihood, confirming that the interaction terms enhanced explanatory power. Adjusted predicted probabilities were subsequently extracted from Model 2 to visualise the interaction and illustrate how screening intention varied jointly by knowledge level and awareness.

Missing data were evaluated prior to modelling. There were no missing variables in our dataset. Multicollinearity was assessed using variance inflation factors (VIFs), all of which were below 2.5, indicating no problematic collinearity. We also screened contingency tables for sparse cells; income categories were collapsed only when necessary to maintain adequate expected counts and preserve conceptual integrity.

### Model diagnostics and sesnitivity analysis

Influence diagnostics, including standardised Pearson and deviance residuals, Δβ values, and Δχ^2^ statistics, were reviewed to identify influential observations. No cases required removal. To assess the potential influence of knowledge categorization on statistical inference, sensitivity analyses were undertaken using alternative specifications of the knowledge variable. Specifically, the knowledge index was re-analysed using a dichotomous classification (lower versus higher knowledge) and model estimates were compared with those obtained from the original three-category specification (low, moderate, and high knowledge).

In addition, outcome classification was evaluated by re-estimating a binary logistic regression model (screened versus not screened) and by examining alternative treatment of respondents who were uncertain about future screening intention. Coefficient stability was further assessed using 1,000 bootstrap replications. Across these analyses, the direction and statistical interpretation of the associations between knowledge, awareness, and screening behaviour remained substantively unchanged, suggesting that the findings were not driven by a particular categorization scheme.

## Results

### Screening uptake

**Fig 1:**
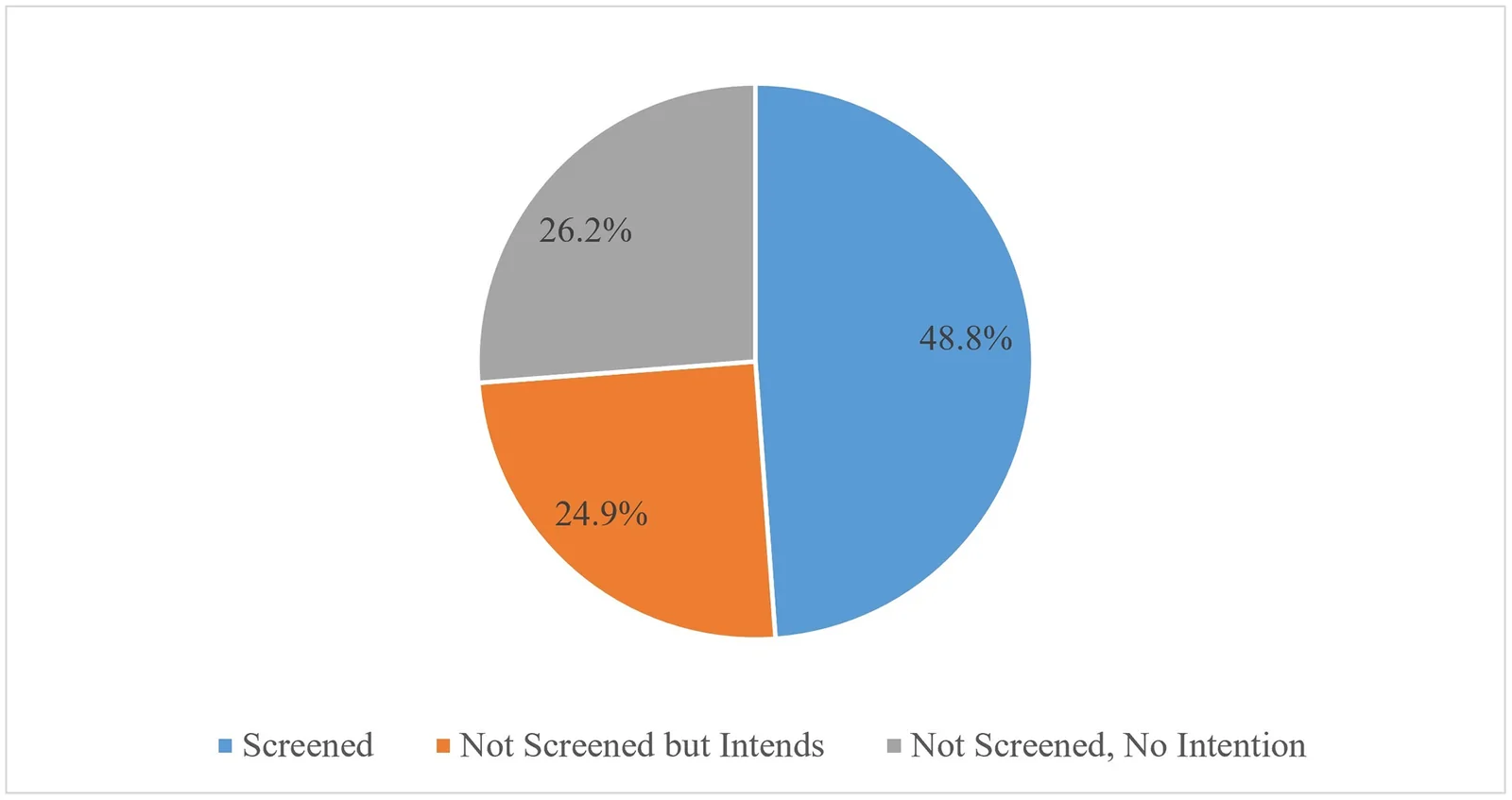
Cervical cancer screening uptake among women aged 25–49 years (n = 385).

Among 385 women, 188 (48.8%) had ever been screened, 96 (24.9%) had not been screened but intended to, and 101 (26.2%) neither had been screened nor intended to (Fig 1). These three categories formed the dependent variable for all subsequent multinomial modelling.

### Knowledge distribution and awareness

**Fig 2:**
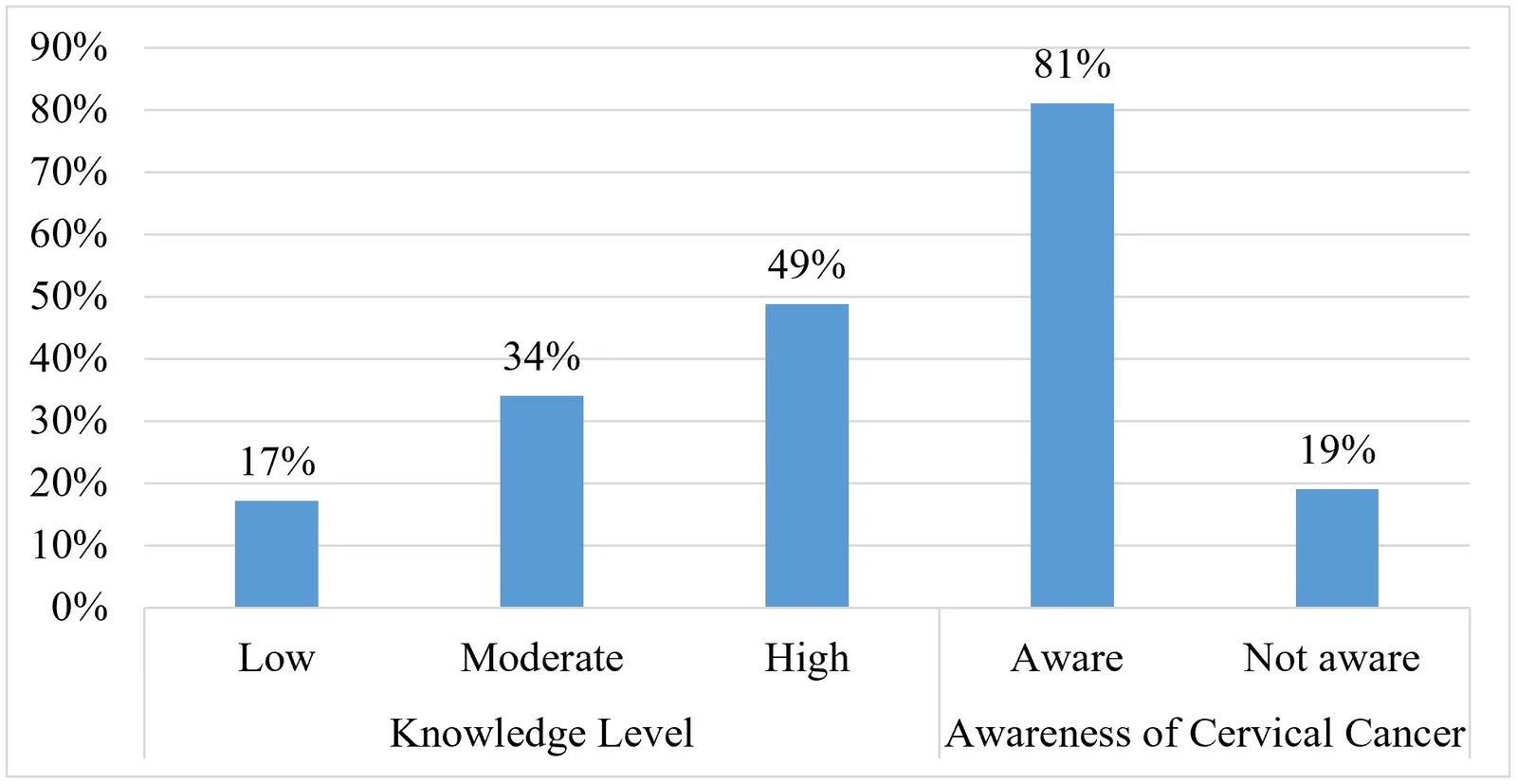
Knowledge-level and awareness distribution among respondents (n = 385).

Knowledge levels were unevenly distributed: Low knowledge (66/385; 17.1%), Moderate (131/385; 34.0%), and High (188/385; 48.8%). Fig 2 shows awareness of cervical cancer was high with 312 (81%) aware, while 73 (19%) were not.

### Association between knowledge and screening behaviour

Knowledge level showed a graded association with screening uptake (χ^2^(4)=58.780, p < 0.001; Cramer’s V = 0.276). Only 18.2% of women with *low* knowledge had been screened, compared with 58.8% among those with *moderate* knowledge and 52.7% among those with *high* knowledge. Conversely, *no intention* was highest among low-knowledge women (62.1%).

Awareness showed a trend toward higher screening (aware 51.3% vs not-aware 38.4%), though the association approached marginal significance (χ^2^(2)=5.203, p = 0.074) (Table 2).

**Table 2:** Screening uptake by knowledge level and awareness (n = 385).

Predictor	Category	Screened (%)	Intends (%)	No Intention (%)	χ^2^ (df)	p-value
Knowledge Level	Low	18.2	19.7	62.1	58.780 (4)	<0.001
	Moderate	58.8	20.6	20.6		
	High	52.7	29.8	17.6		
Awareness	Yes	51.3	22.8	26.0	5.203 (2)	0.074
	No	38.4	34.2	27.4		

### Multivariable models of knowledge and awareness

#### Main-effects model.

The multinomial model with knowledge, awareness, and selected covariates fitted the data well (χ^2^(18)=81.34, p < 0.001; −2LL 645.30; Nagelkerke R^2^ = 0.217). Low knowledge was strongly associated with higher odds of belonging to the “No Intention” group relative to the “Screened” group (AOR = 13.091, 95% CI: 5.526–31.013). Awareness was associated with higher odds of belonging to the “Intends” group relative to the “Screened” group (AOR = 2.270, 95% CI: 1.150–4.483), while lower income was associated with lower odds of belonging to the “Intends” group relative to the “Screened” group (AOR = 0.682, 95% CI: 0.493–0.944) (Table 3).

**Table 3:** Main-effects multinomial regression model for screening uptake (n = 385).

Predictor	Category	AOR	95% CI	p-value
Knowledge (Low)	No Intention vs Screened	13.091	5.526–31.013	<0.001
Awareness (Yes)	Intends vs Screened	2.270	1.150–4.483	0.018
Age	Intends vs Screened	1.307	1.005–1.698	0.046
Income (Low)	Intends vs Screened	0.682	0.493–0.944	0.021

#### Interaction model — Knowledge versus awareness effects on screening behaviour.

In the interaction model, including the Knowledge versus Awareness terms significantly improved model fit (χ^2^(22) = 95.65, p < 0.001; −2LL = 630.99; Nagelkerke R^2^ = 0.251) (Table 4).

**Table 4:** Interaction model (Knowledge versus Awareness) predicting screening uptake (n = 385).

Interaction Term	AOR	95% CI	p-value
Awareness (Yes)	10.123	2.752–37.230	<0.001
Low Knowledge × Awareness	0.024	0.003–0.219	0.001
Moderate Knowledge × Awareness	0.190	0.038–0.956	0.044

Relative to the screened group, awareness was associated with higher odds of belonging to the “Intends” group among women with high knowledge (AOR = 10.123, 95% CI: 2.752–37.230), but this association varied by knowledge level.

The Low Knowledge versus Awareness interaction term (AOR = 0.024, 95% CI: 0.003–0.219) and Moderate Knowledge versus Awareness interaction term (AOR = 0.190, 95% CI: 0.038–0.956) indicate that the association between awareness and belonging to the “Intends” group relative to the “Screened” group was substantially weaker among women with low or moderate knowledge than among women with high knowledge. The positive awareness effect was strongest among women in the high-knowledge reference group. These findings indicate that the association between awareness and screening behaviour varies according to women’s level of knowledge.

### Predicted-probability visualizations

**Fig 3:**
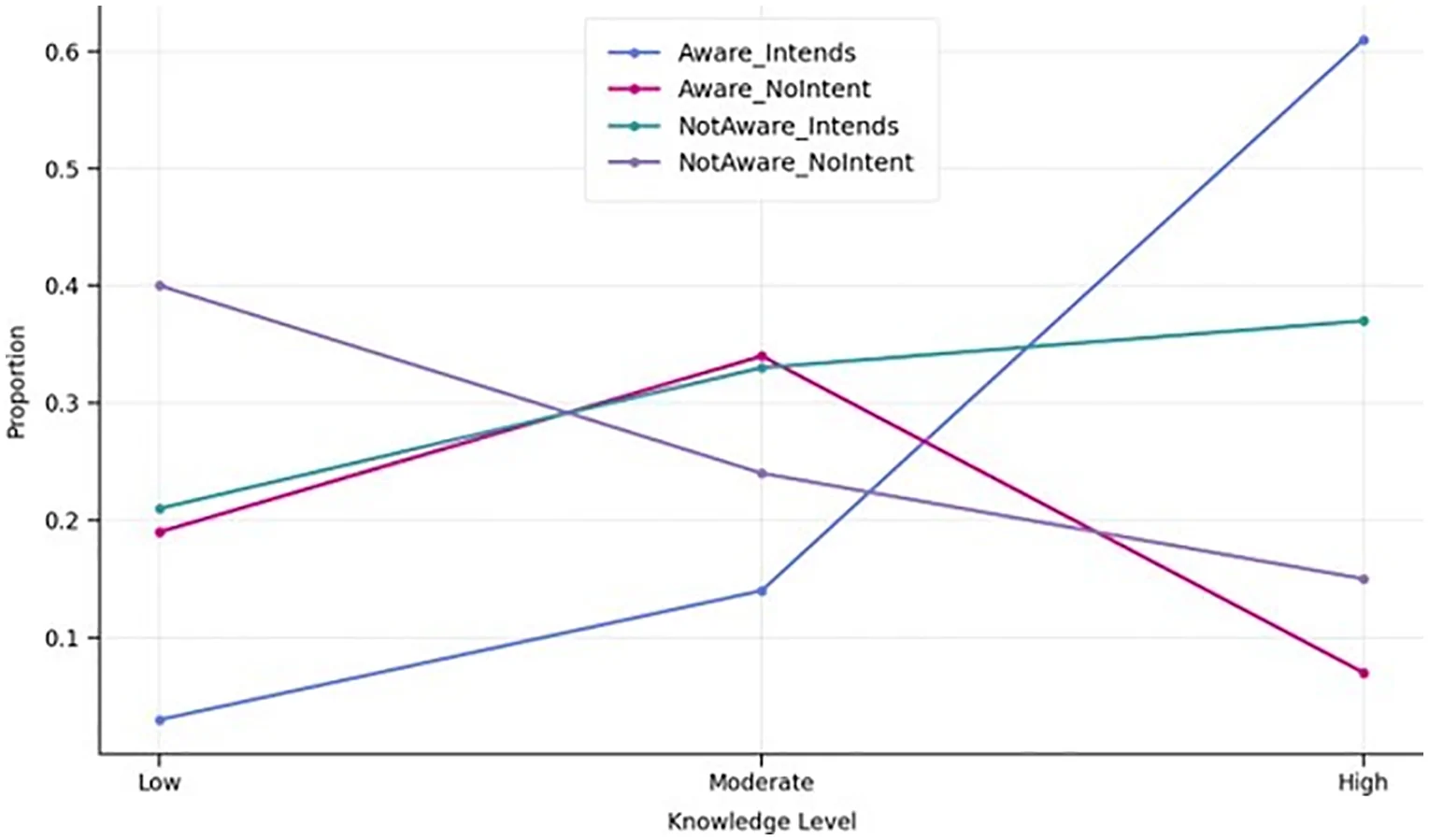
Predicted probability of intention to screen versus knowledge versus (n = 385).

Predicted probabilities from the interaction model further illustrated the knowledge-dependent relationship between awareness and screening intention. As shown in Fig 3, among women who were aware of cervical cancer, the probability of intending to screen was 0.03 (3%) at low knowledge, increased to 0.14 (14%) at moderate knowledge, and rose sharply to 0.61 (61%) at high knowledge.

Among unaware women, intention increased gradually with knowledge, from 0.21 (21%) at low knowledge to 0.33 (33%) at moderate knowledge and 0.37 (37%) at high knowledge. These patterns suggest that the association between awareness and screening intention differs substantially across knowledge levels, with the strongest intention observed among aware women with high knowledge.

## Discussion

This study provides clear evidence that cervical cancer screening behaviour in an urban informal settlement is strongly shaped by the depth of women’s actionable knowledge rather than general awareness alone. Screening uptake was relatively high compared with other LMIC informal settlement contexts, yet nearly two-thirds of women with low knowledge neither screened nor intended to screen. The multinomial and interaction models demonstrated that low knowledge independently predicted non-intention and, critically, that the effect of awareness varied according to knowledge level. Awareness was associated with lower screening intention among women with limited knowledge but was associated with higher intention among women with greater knowledge. These findings suggest that knowledge modifies the behavioural influence of awareness and highlight the importance of actionable understanding in translating information exposure into preventive action [7,8].

The study advances scientific understanding by demonstrating that awareness and knowledge do not operate independently in influencing cervical cancer screening behaviour. While previous African studies consistently show that women with greater knowledge are more likely to screen [7,8], most studies have treated awareness and knowledge as additive predictors. Our findings suggest that the behavioural influence of awareness depends on the depth of women’s understanding of cervical cancer prevention and screening. This helps explain why awareness levels may remain high while screening uptake remains low in many informal settlement settings.

Conceptually, these findings are consistent with the Health Belief Model (HBM), which proposes that preventive health behaviours are influenced by perceived susceptibility, perceived severity, perceived benefits, perceived barriers, self-efficacy, and cues to action. In the present study, actionable knowledge may strengthen perceptions of susceptibility and benefits while improving confidence in the ability to seek screening services, thereby enabling awareness to function as a meaningful cue to action. These pathways align with the Health Belief Model, which posits that preventive health behaviours are shaped by individuals’ perceptions of risk, benefits, barriers, and their confidence in taking action.

The observed knowledge-dependent effects may be particularly important in populations with lower educational attainment. Women with limited formal education may face greater challenges in understanding health information, interpreting risk messages, and navigating available screening services. In such settings, awareness generated through community campaigns may not necessarily translate into actionable knowledge unless information is communicated in simple, culturally appropriate, and practically relevant ways. This may partly explain why high awareness levels do not always correspond to high screening uptake in underserved populations.

Interpretation of the Knowledge–Awareness Interaction suggests that awareness without adequate knowledge may generate confusion, uncertainty, or fear, consistent with regional qualitative studies reporting misconceptions and low self-efficacy when women receive incomplete information about cervical cancer and screening [6,7]. In this context, general awareness may increase perceived severity without strengthening self-efficacy or perceived benefits, potentially leading to avoidance rather than preventive action. Women with higher knowledge, by contrast, may be better able to interpret personal risk, understand the benefits of screening, and navigate available services. From an HBM perspective, knowledge therefore appears to strengthen the cognitive mechanisms through which awareness influences screening behaviour. The rise in intention to screen among highly knowledgeable women further supports the idea that knowledge acts as a cognitive “switch” converting awareness into motivation.

These findings complement ongoing HPV vaccination efforts in Kenya by pointing to the continued importance of effective health education and screening promotion among adult women. While HPV vaccination is expected to reduce future cervical cancer risk, screening remains a critical prevention strategy for women currently within the recommended screening age range and for women who may not have benefited from vaccination programs [13]. Strengthening knowledge and awareness across both vaccination and screening initiatives may therefore contribute to achieving national and global cervical cancer elimination targets [10,11,13].

These findings have important implications for practice. Because the study focused on women aged 25–49 years, the primary target population for cervical cancer screening under Kenya’s national screening guidelines, the findings have direct relevance for current screening policy and program implementation. Community Health Volunteers (CHVs), maternal–child health (MCH) platforms, and facility-based providers should prioritise “actionable knowledge scripts” rather than general awareness campaigns. These scripts should include: the purpose of screening, the correct age range, what happens during the procedure, its safety, and the benefit of early detection. Prior evidence shows that clarity and understandability of information are major predictors of uptake [7]. These considerations can be relevant in settings where educational attainment is low. Screening interventions should therefore move beyond message exposure alone and incorporate educational approaches that strengthen comprehension, self-efficacy, and informed decision-making among women with varying literacy and education levels.

Similarly, frequent interactions with providers such as those seen among women on long-term ART enhance uptake by improving comprehension [8]. Embedding short, structured messages during routine CHV home visits, immunisation days, antenatal visits, and child welfare clinics may strengthen actionable understanding and improve the likelihood that awareness translates into screening intention and uptake.

Programmatic implications are equally substantial. County and national screening programs should reorient strategies from high-volume mass awareness to targeted, depth-focused knowledge interventions. Evidence from Côte d’Ivoire and Malawi shows that even when awareness exceeds 90%, screening uptake often remains below 60% [5,6,8]. This suggests diminishing returns from generic awareness efforts and supports reallocating resources toward personalised, comprehension-oriented communication. Policy frameworks should mandate minimum knowledge competencies for CHVs, provide standardised educational tools aligned with national guidelines, and incorporate knowledge assessment into community outreach monitoring. Additionally, recognising that low-income and less-educated women consistently exhibit lower screening rates across SSA, programs should prioritise tailored approaches for these groups, ensuring equitable behavioural impact.

This study has notable strengths. First, it applies a multinomial outcome that distinguishes between behaviour, readiness, and resistance yielding more policy-relevant insights than conventional binary analyses. Second, the interaction model provides one of the first empirical tests of knowledge-contingent awareness effects in an informal settlement setting. Third, extensive sensitivity analyses confirmed stability of the interaction effect across alternative specifications.

Nevertheless, several limitations warrant consideration. The cross-sectional design limits causal inference; however, the consistency of associations with existing literature enhances interpretive confidence. Knowledge may have been misclassified due to one reverse-scored item, though sensitivity tests showed minimal impact on estimates. Self-reported screening status may be subject to recall or social desirability bias, as observed in other regional studies [7]. We also note that while the study isolates knowledge-related determinants, unmeasured system-level factors service availability, provider behaviour, or facility readiness may also shape uptake and should be explored in future mixed-methods research. Finally, the findings are most directly applicable to women aged 25–49 years, the population group targeted by national screening guidelines. Consequently, the results may not be fully generalizable to younger or older women outside the recommended screening age range.

In addition, knowledge was analysed using categorized index scores to facilitate interpretation and interaction modelling. Although categorization may reduce statistical efficiency and potentially increase sensitivity to selected cut-points, sensitivity analyses using alternative knowledge classifications and bootstrap validation produced substantively similar findings. Nevertheless, future studies using continuous knowledge measures may provide a more precise assessment of the relationship between knowledge and screening behaviour.

Overall, this study provides novel and mechanistic evidence demonstrating that screening behaviour in informal settlements is governed not by exposure to messages but by the depth of women’s actionable knowledge. By demonstrating that the behavioural influence of awareness depends on women’s level of actionable knowledge, the study offers an explanatory pathway for persistent intention–action gaps and identifies a theoretically grounded target for intervention.

## Conclusion

This study shows that cervical cancer screening behaviour in an informal settlement is shaped not simply by awareness but by the depth and quality of women’s actionable knowledge. Low knowledge was associated with reduced screening intention, and the influence of awareness differed substantially across knowledge levels. These findings suggest that awareness alone may be insufficient to promote screening behaviour unless accompanied by accurate and actionable understanding of cervical cancer prevention and screening. Strengthening knowledge content within community and facility-based health communication strategies may therefore represent an important pathway for improving cervical cancer screening uptake in underserved populations.

### Strengths and limitations

#### Strengths.

- The study used a multinomial modelling approach that distinguished between screening, intention, and non-intention, providing deeper behavioural insights missing in most African screening studies.
- Interaction modelling enabled examination of how awareness and knowledge jointly influence screening behaviour, addressing gaps in previous research where these factors were typically treated as independent predictors.

#### Limitations.

- The cross-sectional design limits causal inference, a common constraint in regional screening studies
- Screening status and knowledge were self-reported and may be affected by recall or social desirability bias, as documented in similar comparable population-level studies.
- Knowledge was analysed using categorized index scores to facilitate interpretation and interaction modelling. Although sensitivity analyses supported the robustness of the findings, future studies using continuous knowledge measures may provide a more precise characterization of knowledge-related effects on screening behaviour.

**What Is Known**

- Cervical cancer screening uptake in many African settings remains low despite high levels of awareness, and studies consistently show that clarity and accuracy of information strongly influence screening behaviour.
- Women who receive understandable, actionable knowledge are significantly more likely to participate in screening than those exposed only to general awareness messages.

**What This Study Adds**

- This study demonstrates that the relationship between awareness and cervical cancer screening behaviour varies according to women’s level of knowledge.
- It reveals a behavioural reversal, awareness reducing intention at low knowledge levels explaining why high-awareness in informal settlements still experience low uptake.
- It demonstrates that knowledge depth, rather than awareness volume, is the key leverage point for improving cervical cancer screening behaviour.

## Supporting information

S1 Data HH Survey. (XLSX)

## Abbreviations

ART: Antiretroviral therapy
CDC: Centers for Disease Control
DNA: Deoxyribonucleic acid
HIV: Human immunodeficiency virus
HPV: Human papillomavirus
LMICs: Low- and middle-income countries
MPHIA: Malawi Population-Based HIV Impact Assessment
PEPFAR: President’s Emergency Plan for AIDS Relief
VIA: Visual inspection with acetic acid
WHO: World Health Organization
WLHIV: Women living with HIV
