# Supplementary material for: Awareness, knowledge, and cervical cancer screening behaviour in an urban informal settlement: Evidence from multinomial and interaction models
Source: PLOS Glob Public Health. 2026 Aug 21;6(8):e0007013. doi: 10.1371/journal.pgph.0007013 (PMC13496422; doi:10.1371/journal.pgph.0007013)
Supplement: Supplementary file 1 [file pgph.0007013.s003.docx]

**AUTHORS’ CORRECTION MATRIX | RESPONSE TO REVIEWERS**

Manuscript ID: **PGPH-D-26-00582** | Title: “**A Knowledge-Threshold Mechanism for Cervical Cancer Screening in Urban Informal Settlements: A Multinomial and Interaction Modeling Analysis”**

Dear Editor and Reviewers,

We sincerely thank the Editor and Reviewers for their thoughtful and constructive comments. We have carefully revised the manuscript and addressed all concerns raised. Detailed responses pointing to the specific actions and sections in the track-change version of the revised manuscript are provided in Tabular format below.

| Comment | Response | Action Taken in Manuscript | Page(s) |
| --- | --- | --- | --- |
| Editor Comment 1: One of the authors (Alfred Owino) appears as a member of the Ethics Review Committee. Please explain how this was handled. | Mount Kenya University Ethics Review Committee policy requires members to recuse themselves from review, deliberation, and approval of protocols in which they are investigators, supervisors, collaborators, or otherwise involved. Dr. Alfred Owino did not participate in the review, discussion, or approval of this protocol. The application underwent independent review and approval by other committee members in accordance with institutional ethical procedures. | Clarification provided in the Response to Reviewers letter and ethical documentation submitted. | Review Response Letter |
| Editor Comment 2: Please provide additional details regarding participant consent.  “ Please provide additional details regarding participant consent. In the ethics statement in the Methods and online submission information, please ensure that you have specified (1) whether consent was informed and (2) what type you obtained (for instance, written or verbal, and if verbal, how it was documented and witnessed). If your study included minors, state whether you obtained consent from parents or guardians. If the need for consent was waived by the ethics committee, please include this information.” | The Ethics and Participant Protections section has been revised to provide additional detail regarding the consent process. We now explicitly state that written informed consent was obtained from all participants prior to data collection. Participants received study information in either English or Kiswahili and were informed about the study objectives, procedures, potential risks and benefits, confidentiality protections, and their right to decline participation or withdraw at any time without penalty. We have also clarified that only woman aged 25–49 years were eligible for participation; therefore, no minors were enrolled in the study and parental consent was not applicable. In addition, the ethics statement was revised to distinguish ethical approval, research permit authorization, and administrative approval in accordance with Kenyan research governance requirements. | Ethics and Participant Protections section revised to provide details on written informed consent procedures and clarify ethical approval, research permit, and administrative approval processes. | Page 15-16 |
| Editor Comment 3: Please provide separate figure files in TIFF or EPS format. | All figures have been converted to journal-compliant TIFF format and submitted separately according to PLOS requirements. | Separate figure files uploaded. | Submission files |
| Editor Comment 4: Data availability statement does not meet PLOS requirements.  “In the online submission form, you indicated that "The dataset supporting the conclusions of this article is available from the corresponding author upon reasonable request, in accordance with ethical approval conditions.".  All PLOS journals now require all data underlying the findings described in their manuscript to be freely available to other researchers, either  1. In a public repository,  2. Within the manuscript itself, or  3. Uploaded as supplementary information.” | We appreciate this observation. The data availability statement has been revised. A de-identified dataset has been provided as Supporting/supplementary Information to ensure compliance with PLOS data-sharing requirements while protecting participant confidentiality. | Data Availability Statement revised and de-identified dataset uploaded as Supporting Information  Data file (excel) uploaded | Page 29 |
| Reviewer 1 Comment 1: How was the questionnaire validated? | Additional information has been added describing the questionnaire validation process. The instrument was reviewed by two public health experts to assess content validity, clarity, and relevance. It was subsequently pretested among 25 women in Makongeni Informal Settlement, a community comparable to the study setting. Feedback from the pretest informed revisions to question wording, sequencing, and cultural appropriateness. Internal consistency of the knowledge scale was assessed using Cronbach’s alpha and demonstrated acceptable reliability (α = 0.81). | Expanded questionnaire validation description to include expert review, pretesting procedures, questionnaire refinement, and reliability assessment. | Page 10 |
| Reviewer 1 Comment 2: What was the basis for categorizing the knowledge variable and what informed the categories?” | Additional clarification has been added to the Methods section. Knowledge was measured using a five-item index (score range 0–5) reflecting understanding of cervical cancer prevention, screening, and early detection. Categories were defined a priori to represent meaningful levels of actionable knowledge: low knowledge (0–1 correct responses), moderate knowledge (2–3 correct responses), and high knowledge (4–5 correct responses). The categorization was intended to distinguish women with limited, partial, and comprehensive understanding while maintaining adequate sample sizes within categories for multinomial and interaction modelling. To assess whether findings were sensitive to this categorization, additional sensitivity analyses using alternative knowledge specifications were conducted and produced substantively similar results. | Expanded the description of knowledge categorization and rationale in the Measures and Operational Definitions section and cross-referenced supporting sensitivity analyses. | Pages 11–12 |
| Reviewer 1 Comment 3: Why was age limited to 25–49 years? | Additional justification has been added to the manuscript. Women aged 25–49 years were selected because this age group constitutes the primary target population for cervical cancer screening under Kenya National Cancer Screening Guidelines and aligns with WHO recommendations for cervical cancer prevention and screening in resource-constrained settings. The age range was therefore chosen to ensure that the study focused on women for whom screening services are routinely recommended and most relevant from a public health perspective. | Justification for the selected age range added to the study population and eligibility criteria section and reflected in the discussion of study scope and applicability. | Pages 9, 15, and 22–24 |
| Reviewer 1 Comment 4: How might findings have been affected in a setting with low education levels? | We agree that educational attainment may influence how women interpret and act upon health information. Additional discussion has been added to highlight that lower educational attainment may limit comprehension of cervical cancer prevention messages, thereby reducing the likelihood that awareness translates into actionable knowledge and screening behaviour. We further note that awareness campaigns relying primarily on information exposure may be less effective in populations with lower educational attainment unless accompanied by clear, culturally appropriate, and actionable educational content. | Discussion expanded to consider the potential influence of educational attainment on awareness, knowledge acquisition, and screening behaviour, and to discuss implications for health communication strategies in underserved populations. | Pages 21 and 23 |
| Reviewer 1 Comment 5: What HPV vaccination programs are currently ongoing in Kenya? | Additional context has been added to the Introduction and Discussion sections describing Kenya's national HPV vaccination program, including routine vaccination of adolescent girls through school- and facility-based delivery platforms. We also highlight ongoing efforts to improve vaccine uptake through community mobilization, integration with adolescent health services, and public awareness initiatives. This information provides important context for understanding cervical cancer prevention efforts and the continued importance of screening among eligible women. | introduction and Discussion sections expanded to provide context on Kenya's HPV vaccination program and its implications for cervical cancer prevention and screening.  Pages: | Page 7 and Page 22 |
| Reviewer 1 Comment 6: Categorizing knowledge may inflate type I error. How was this addressed? | We acknowledge that categorization of knowledge scores may result in some loss of information, reduced statistical efficiency, and potential sensitivity to selected cut-points. In this study, categorization was undertaken to facilitate interpretation of knowledge-related differences in screening behaviour and to support multinomial interaction modelling.  To evaluate the robustness of the findings, we conducted several sensitivity analyses. These included: (i) re-estimating the model using a binary outcome (screened versus not screened) instead of the multinomial outcome; (ii) reclassifying respondents who reported being uncertain about future screening intention either as missing observations or within the no-intention category; (iii) dichotomizing knowledge scores into lower versus higher knowledge categories using an alternative classification scheme; and (iv) validating coefficient estimates through 1,000 bootstrap replications. Across these analyses, the direction and interpretation of the key associations, including the interaction between knowledge and awareness, remained substantively unchanged. These findings suggest that the reported results were not dependent on a single categorization approach. | We have expanded the Methods section to describe these robustness checks and added a limitation acknowledging the potential loss of information associated with categorization. We also note that future studies using continuous modelling approaches may provide additional insights into the relationship between knowledge and screening behaviour. | Pages 14–15 and 24 |
| Reviewer 1 Comment 7: What happened to participants identified as having low knowledge? | Although individual knowledge levels were not disclosed or used to determine participant treatment during data collection, all participants received standardized cervical cancer health education immediately following completion of the interview, irrespective of their knowledge level or screening status. Information included key messages on cervical cancer prevention, recommended screening age, available screening services, and the importance of early detection. This approach ensured that participation was associated with an equitable educational benefit while avoiding differential treatment based on study responses. | Participant follow-up procedures were clarified to indicate that all participants received standardized post-interview cervical cancer health education and information on available screening services.  **Page 10 (Data Collection)** explains *what was done operationally while*  **Page 15 (Ethics Section)** explains *why it was done ethically*. | Pages 10 and 16 |
| Reviewer 1 Comment 8: Interpretation of odds ratios is confusing. | We agree that multinomial odds ratios must be interpreted relative to the reference outcome category. In our analyses, “Screened” was specified as the reference category. The Results section has therefore been revised throughout to ensure that adjusted odds ratios are interpreted relative to the appropriate comparison category rather than as absolute increases or decreases in screening behaviour. Language that could be interpreted as implying linear effects or direct probability changes has been removed and replaced with odds-based interpretations consistent with multinomial logistic regression methodology. | Results section revised throughout to clarify multinomial odds ratio interpretation relative to the reference outcome category (“Screened”). Table descriptions and narrative interpretations were aligned with multinomial modelling conventions. | Pages 13, 17–20 |
| Reviewer 1 Comment 9: The title suggests a threshold should be estimated, but no specific threshold was identified.  *“Overall, if one was to speak to the title of this manuscript, folks would expect the authors to report the threshold of knowledge at which we are likely to see cervical cancer screening in this community. This would require looking at knowledge as a continuous variable. As it is now, it's a bit difficult to appreciate the main aim of this work versus what has been reported and the way the analysis was conducted.”* | We agree that the original title may have implied identification of a precise knowledge threshold, whereas our analysis was designed to evaluate the interaction between awareness and knowledge categories rather than estimate a specific threshold value using a continuous knowledge measure. To better align the title with the study design, analytical approach, and findings, we have revised the title to: **“Awareness, Knowledge, and Cervical Cancer Screening Behaviour in an Urban Informal Settlement: Evidence from Multinomial and Interaction Models.”** In addition, we have clarified in the Introduction and Discussion that our findings demonstrate knowledge-dependent differences in the effect of awareness on screening behaviour rather than a mathematically estimated threshold. We also acknowledge that future studies using continuous modelling approaches may be useful for identifying specific threshold values. | Title revised to reflect the suggestions based on the reviewers’ note; clarifications added to the Introduction, Discussion, Limitations and Conclusion sections. | Title, Pages 1–3, 7–8, 19–25 |
| Reviewer 2 Comment: Please ensure that the Health Belief Model is appropriately cited in the discussion section. | The Discussion section has been revised to more explicitly situate the findings within the Health Belief Model (HBM). We now explain how actionable knowledge may strengthen perceptions of susceptibility, benefits of screening, self-efficacy, and responsiveness to cues to action, thereby facilitating cervical cancer screening behaviour. Appropriate citations to the Health Belief Model literature have been added to support this interpretation. | Discussion section expanded to explicitly link study findings to key Health Belief Model constructs and relevant citations added. | Pages 21–22 |

**Authors’ Comment:**

We believe these revisions have improved manuscript and have addressed all concerns raised by the Editor and Reviewers to meet the journal requirements. We thank the Editor and Reviewers for their valuable feedback and consideration.
